# Perceptions of patients with end-stage kidney disease (ESKD) and their informal caregivers on palliative care as a treatment option: a qualitative study

**DOI:** 10.1186/s12904-020-00640-y

**Published:** 2020-08-20

**Authors:** Catherine Sarfo-Walters, Edward Appiah Boateng

**Affiliations:** 1 Department of Nursing, Garden City University College, Kumasi, Ghana; 2 Department of Nursing, Kwame Nkrumah University of Science and Technology, Kumasi, Ghana

**Keywords:** End-stage kidney disease, Palliative care, Conservative management, Haemodialysis, Ghana, Africa

## Abstract

**Background:**

Palliative care is increasingly becoming an accepted treatment choice for many individuals diagnosed with end-stage kidney disease (ESKD). Yet, its utilisation is non-existent in many lower- and middle-income countries (LMICs). This study explored the perceptions of individuals with ESKD and their informal caregivers on palliative care as a treatment option for the disease in Ghana.

**Methods:**

This was a phenomenological study, with an in-depth analysis of data collected from nine individuals with ESKD and six informal caregivers through individual, face-to-face semi-structured interviews. The study was conducted in two renal centres within the Kumasi metropolis, Ghana among individuals with ESKD seeking care from both renal centres and their informal caregivers.

**Results:**

Three main themes were derived from this study – motivation for initiating haemodialysis, facing realities of haemodialysis, and considering palliative care. Participants felt that haemodialysis (HD) was not meeting their health expectations and demonstrated a general willingness to utilise palliative care if it would reduce suffering.

**Conclusions:**

This study has shown that individuals with ESKD or their informal caregivers would consider palliative care services, if available. It paves the way for discussions about palliative care for ESKD to begin across renal centres within Ghana and other similar settings. Exploring perspectives of clinicians in such settings could inform strategies on how to implement palliative care for ESKD management in such settings.

## Background

Palliative care for ESKD has been defined as “a transition from a conventional disease-oriented focus on dialysis as a rehabilitative treatment to an approach prioritizing comfort and alignment with preferences and goals of care to improve quality of life and reduce symptom burden” [1, 2]. It comprises interventions that focus on slowing the deterioration of kidney function, minimising the risk of adverse events, shared decision-making, active symptom management, detailed communication, including advance care planning, psychological, social and family support, and cultural and spiritual care, but does not include RRT [3–5]. The focus is on improving the quality of life of individuals with ESKD, in line with their values and goals, rather than merely extending their life [4, 6–8]. This is increasingly becoming an accepted treatment choice and is highly recommended for individuals in whom renal replacement therapy (RRT) may offer little or no significant difference in terms of survival or quality of life [3, 4, 6, 9–14]. Indeed, the World Health Organization [15] classifies ESKD among diseases that would benefit from palliative care.

Individuals with ESKD, cared for in renal centres where palliative care services are well-established, choose palliative care more often than centres without palliative care services [2, 16]. It has been reported that individuals with ESKD would prefer to be given adequate information about the condition and their treatment options, including palliative care, at an earlier stage of the disease [17]. Yet, palliative care for managing ESKD is limited in several countries because of inadequate well-trained palliative care clinicians and poor access to palliative care services [4, 7]. This is, especially, more profound in lower- and middle-income countries (LMICs) where financial, geographical and infrastructural challenges affect the provision of efficient renal services [18–22]. Inadequate information provision and delayed initiation of discussions on palliative care also remain key barriers to its utilisation as a treatment option for ESKD [2, 17, 23, 24]. Informal caregivers of individuals with ESKD, mainly relatives and friends, may also discourage clinicians’ efforts at providing open and honest information about palliative care services [25].

Ghana is noted to have made improvements in the provision of palliative care services, being ranked 8th in Africa [26–28]. However, like in other African countries, palliative care largely focuses on HIV and cancer-related diagnoses [20, 28]. There are no established pathways to palliative care for ESKD management, and the subject of death is rarely broached in discussions between clinicians and patients [18]. This study, therefore, explored the perceptions of individuals with ESKD and their informal caregivers on palliative care as a treatment option of the disease in Ghana.

## Methods

### Research setting

Ghana has inadequate renal services, with fifteen renal centres all located in five out of the sixteen administrative regions of the country [29]. These centres are managed by a few nephrologists, nephrology trainees, consultant physicians, specialist physicians, medical officers and, largely, by nurses who are trained-on-the-job [18, 29]. Payment for the treatment of ESKD is mainly made out-of-pocket by patients or their significant others in Ghana as the National Health Insurance Scheme (NHIS) does not cover the treatment costs [18, 22, 29]. This results in only a few patients with ESKD being able to initiate and sustain treatment, and the increased tendency of reducing the frequency of dialysis, withdrawing from treatment and/or pursuing other forms of treatment outside biomedicine [18, 30].

This study was conducted in two renal centres that offer standard conservative care to individuals with ESKD who opt for it, including the treatment of anaemia, management of hypertension, pain as well as dietary and fluid management approaches, similar to other established renal centres [22, 31]. Although there are a few specialist palliative care services in the country, these are largely utilised by patients with advanced types of cancers and largely inaccessible to a majority of the citizenry owing to costs [22, 28].

### Study design, sampling and data collection

This was a qualitative study, utilizing the phenomenological approach as it focussed on the lived experiences of individuals with ESKD and their informal caregivers and their perceptions about palliative care [32, 33]. The study targeted individuals with ESKD receiving haemodialysis at the two renal centres as well as their informal caregivers. A purposive sampling technique was employed for this study. After administrative approval was given, CS-W was introduced to eligible participants in both renal centres by the respective nurses-in charge to discuss the objectives of the study and their possible inclusion. Those who agreed confirmed their participation by signing or thumb-printing the consent form before being interviewed by CS-W. All those who were approached agreed to be part of the study.

Data for this study was collected through individual, face-to-face, semi-structured interviews. Each renal centre provided an office where the interviews were conducted, with only CS-W and the interviewee present in the office during each interview session. Two separate written interview guides were employed, one for the individual with ESKD and the other for informal caregivers to ensure comprehensive accounts from each interview session. Each interview guide was evaluated after the first session to ensure their accuracy, understandability, and appropriateness for this study, and to serve as a pilot test. There were no repeat interviews. Interview sessions lasted between 30 and 90 min and each was recorded using a digital audio-recording device. Field notes were written after each interview session to provide detailed observations and mannerisms that were not captured through the audio-recording. All audio-recordings were transcribed for data analysis. Two of the interview sessions were conducted in Twi, a popular Ghanaian dialect, and these were translated by CS-W. EAB and an independent researcher who are both fluent in the Twi and English languages verified the accuracy of the translation.

### Data processing and analysis

All interviews were transcribed after each session. NVivo 10 software was used to manage and organize the data. Data collection and analysis occurred simultaneously, with the Colaizzi method [34, 35] guiding the analysis. All transcripts were proofread while listening to the audio recordings to ensure accuracy. Eleven participants agreed to look at their respective transcripts for comments and possible corrections and deletion of personal information – all were happy with the transcript and had no changes to be made. All transcripts were read multiple times and coded independently by CS-W and EAB. The field notes were also read to aid the contextualisation of various participant accounts during analysis. Significant statements about participants’ experiences with management of ESKD and their perceptions of palliative care were extracted from their respective transcripts and meanings formulated from these statements. Each participant’s experiences and perceptions were studied separately and then compared across transcripts. Both authors examined the data and used codes, patterns, and themes to classify them, meeting frequently to discuss emerging themes and agreeing on those that needed further exploration in subsequent interviews. These meetings continued until both authors agreed that themes had been adequately explored for the purpose of the study and ended data collection [36].

### Ethical considerations

All participants gave their consent, either by signing or thumb-printing an informed consent form before participating in the study. Ethics approval (ID No: UCCIRB/CHAS/2015/105) was obtained from the Institutional Review Board of the University of Cape Coast, Ghana before data collection commenced. Participants were assured of confidentiality and anonymity, and their names and other personal identifiers have been replaced with pseudonyms in this paper to ensure this.

### Trustworthiness

Data was collected from two renal centres as well as from two sources as a way of achieving triangulation and, in effect, ensuring credibility [37]. Also, member checks allowed participants to correct errors to ensure credibility [38, 39]. In this study, participants who were contacted did not find any errors or need for additional information. Both researchers coded and analysed the data independently, meeting to compare outcomes and resolve differences. Interpretation of data was supported by direct quotes from participants to enhance credibility and confirmability of the study findings [40–42]. Transferability has been enhanced by providing an in-depth description of the population and the context of the study [41]. This report has been guided by the Consolidated Criteria for Reporting Qualitative Research [43].

### Reflexivity

In qualitative studies, the researcher becomes the instrument and is intimately involved in data collection and analysis. It was, therefore, important to reflect upon biases the researcher may have, a term called reflexivity [40]. CS-W has over 10 years’ worth of experience in the management of ESKD. She is a nurse educator and served as a member of the palliative care team in her hospital. EAB has served as a nurse educator in Ghana for 9 years. He has a keen interest in enhancing the quality of life as well as decision-making experiences of individuals with ESKD. These potential biases were taken into consideration while collecting and analysing the data.

## Results

Fifteen participants, comprising nine individuals with ESKD (patient-participants) receiving haemodialysis and six informal caregivers were included in this study. The sample size was determined after achieving data saturation [40]. Seven of the patient-participants were male while the remaining two were female. Duration on haemodialysis ranged from 5 months to 5 years. The youngest patient-participant was 20 years old while the oldest was 65 years old. Four of the six informal caregivers were female, and all were closely related to the patients with ESKD. Details of all participants have been provided in Table 1.

**Table 1:** Characteristics of study Participants

Details of patient participants			
**Name**	**Age range (years)**	**Duration on haemodialysis**	**Comorbidity**
Michael	61–65	5 months	Hypertension, diabetes mellitus
David	18–25	5 months	None
Peter	61–65	3 years	Hypertension, diabetes mellitus
Martha	26–30	3 years	None
Richard	51–55	3 years	Hypertension, diabetes mellitus
Daniel	46–50	3 years	Hypertension
Sandra	36–40	8 months	Hypertension
Paul	66–70	3 years	Hypertension, diabetes mellitus
Raymond	56–60	5 years	Hypertension
**Details of informal caregivers**			
**Name**	**Age (years)**		**Relationship to ESKD patient**
Gloria	51–55		Mother
Margaret	46–50		Mother
Doreen	51–55		Wife
Douglas	31–35		Brother
Paulina	26–30		Sister
Eric	18–25		Nephew

Three main themes were derived from the analysis of the data – motivation for initiating haemodialysis, facing realities of haemodialysis, and considering palliative care. Table 2 provides a summary of these themes and their sub-themes. Each of these three themes has been presented below, with relevant quotations from patient-participants, and from informal caregivers to corroborate statements from patient-participants.

**Table 2:** Themes and sub-themes

Themes	Sub-themes
Motivation for initiating Haemodialysis	Facing life-threatening prognosis
	Mistaken expectations of cure
Facing realities of haemodialysis	Financial demands of haemodialysis
	Becoming a burden to family
	Worsening quality of life
Considering palliative care	Death is inevitable
	Desiring dignified death

### Motivation for initiating haemodialysis

Analysis of data showed that two main factors served as motivation for haemodialysis initiation – facing life-threatening prognosis and mistaken expectations of a cure. The sub-theme ‘facing life-threatening prognosis’ described participants’ expression of a full realisation that ESKD is life-threatening, especially without any form of RRT. This was mainly as a result of the clinical manifestations experienced and information gained from the renal centre. Others were also as a result of the poor prognosis of other patients with ESKD that they had met at the renal centre.

*“It [ESKD] puts you in a state of fear because of the breathlessness, and blood issues [anaemia] – will I wake up the next day?” (Daniel, ESKD patient).*

*“Yes, we think it can cause her death. Many times, she cannot breathe at night. All the time she needs to be transfused. Looking at how emaciated and the suffering she is going through I do not doubt that this will be her cause of death … I will not be surprised” (Gloria, caregiver).*

Some participants also started haemodialysis, hoping that a series of sessions would cure the disease. This mistaken expectation of a cure was the driving force for most of the initial decisions about treatment.

*“I thought it [haemodialysis] was only for a few sessions and I will no longer require the treatment” (Michael, ESKD patient).*

*“Our understanding was that the haemodialysis is going to cure her of [the] symptoms she is experiencing” (Gloria, caregiver).*

### Facing realities of haemodialysis

Participants generally re-examined their health expectations and pondered the worth of haemodialysis, with its associated challenges, after receiving the treatment for some time. The theme ‘facing realities of haemodialysis’ describes this phase for participants. Expectations of haemodialysis were modified to the point where participants now hoped that their clinical manifestations would be as minimal as possible. Unfortunately, many participants felt that these modified expectations were also difficult to achieve. Some patient-participants reported that they had not noticed any improvements in their health status or quality of life even though they had been on haemodialysis for some time.

*“Sometimes I have swollen leg; you can’t know where it comes from … I vomit, I go to the toilet [diarrhoea], and these are abnormal things that will not happen to any normal human being. Even though I’m doing the haemodialysis, these continue to come” (Peter, ESKD patient).*

The burden of living with ESKD, including changes in health status and continuously raising large sums to finance their treatment but with little to no improvements led to feelings of despair among patient-participants. Indeed, they reported using all resources available to them to pursue treatment. Patient-participants also felt that they had become a burden to their significant others because they needed their assistance in performing activities of daily living, visiting the renal centre, or paying for the haemodialysis costs.

*“They are fed up with me, they come with me to haemodialysis but they complain all the time, you see they are tired of following me here and there” (David, ESKD patient).*

Informal caregivers reiterated the concerns about the realities of having their family member on haemodialysis and how onerous it is for the entire family.

*“There are challenges because haemodialysis is costly. Everything is cash-and-carry and you are expected to pay without the National Health Insurance Scheme, not to talk about the cost of the [erythropoietin] injection, transportation and long hours on the machine, it is a worry” (Douglas, caregiver).*

The informal caregivers also corroborated the views of patient-participants that haemodialysis was not offering any improvements in their quality of life or health status.

*“She has never been well. Not a single day will this woman [patient] say ‘I feel well’. After six months of haemodialysis, there are no signs of her getting well. It is very frustrating” (Paulina, caregiver).*

This led to the next theme describing participants’ perceptions regarding death and their prevailing circumstances surrounding treatment with haemodialysis.

### Considering palliative care

Participants perceived palliative care as a means to provide relief from pain and other symptoms, ensuring that death does not come through endless suffering. Participants generally perceived death as an event that befalls all mankind and preferred not to expend all their resources on haemodialysis, cognisant of the inevitability of death and the devastating effect that their continuous utilisation of haemodialysis could have on their family. They also believed that, through palliative care, they could avoid or control suffering before death.

*“I will opt for palliative care if that service is available for me so that I will not suffer but die peacefully” (Michael, ESKD patient).*

Generally, informal caregivers were also of the view that if haemodialysis was not meeting their expectations of improved quality of life for their relatives nor guaranteeing survival, then they would not be reluctant to choose palliative care if the service is available.

*“If we know the person will die eventually, I think she does not need to go through all these and lose [her] life. For me, I think that if the person cannot afford the haemodialysis, palliative care should be an option” (Gloria, caregiver).*

Some patient-participants advocated that palliative care should be implemented and awareness created to enhance choosing it as an alternative or alongside haemodialysis during decision-making on treatment.

*“Yes, in fact, please do that (implement palliative care). Life continues even after death, that’s our belief. So, if I’m not getting well via the haemodialysis and somebody has time to talk to me, discuss my problems and spiritual wellbeing, why not?” (Peter, ESKD patient).*

For relatively younger individuals with ESKD, however, caregivers felt uneasy about being introduced to palliative care. Yet, the belief that death is inevitable made them do all that they could in order not to appear non-supportive of their relative.

*“It [palliative care] can help but can you look at a young man like this one to die? What can we say? If it is the will of God, little can we do but we wish we can support him … in that case if anything happens, you know it is the will of God” (Margaret, caregiver).*

## Discussion

This study aimed at exploring the perceptions of participants on palliative care as a treatment option for ESKD in Ghana. Many participants did not know much about the chronic and potential terminal nature of ESKD at the time of diagnosis and so were expectant of a cure when initiating treatment. Expectations of a cure around the time when treatment of ESKD is initiated has already been reported in Ghana [18] so this was, somewhat, not surprising. However, being on treatment for some time and experiencing symptoms associated with the disease made them perceive death as a real possibility. It is well-known that ESKD is a life-threatening condition and those living with it confront mortality as part of their daily experiences [44], and personal or vicarious experiences of clinical manifestations of ESKD led participants of this study to confront their mortality. Our study found that during such moments, deeper meanings about death were reflected upon. Comments that implied that death is part of life reflect their effort to ‘normalise’ death as a potential outcome of the disease. This did not always suggest acceptance of their prognosis though, especially as they had not had formal discussions with their clinicians about their prognosis and palliative care.

Palliative care is recommended for patients with ESKD in whom RRT would not offer survival benefits or improved quality of life. These are usually the very elderly, those with comorbidities as well as those who initiate RRT within three months of diagnosis as a result of late presentation [3, 4, 6, 9–13]. For such individuals, palliative care ensures that they receive quality care as they near the end of life. Although patients with ESKD are generally young in the Ghanaian setting, late presentation, poor functional status and high mortality rates associated with ESKD imply that many could benefit from palliative care services if this choice was to be available [9, 18, 19, 22, 45, 46]. The treatment choices of the Ghanaian patient with ESKD are limited in so many ways when survival is the expected outcome, unlike in many high-income countries where there is a menu of life-saving treatment choices available to the patient [18, 47, 48]. Despite the modality constraints, the initial drive to preserve life led participants to initiate haemodialysis until they realised that sustainability is nearly impossible or comes with its daunting adjustments.

Studies on palliative care for ESKD report that this treatment choice is made because individuals do not want to burden their families, feel neglected, experience a deterioration of their quality of life, experience acute medical complications, or when they are unsure of good prognosis [7, 44, 49, 50]. The desire not to burden others also seemed to play a role in making individuals living with ESKD in this study consider accepting palliative care. However, consideration for palliative care mainly stemmed from realising that there is no certainty of a good prognosis, coupled with a general deterioration of their quality of life. The impact of haemodialysis on individuals’ or their families’ finances also had a stronger role to play in their search for a different treatment option. Indeed, there are instances where comprehensive conservative care becomes the only option in the face of resource constraints or limited access to renal replacement therapy [5, 14]. However, comprehensive conservative care of patients with ESKD must not be perceived as a low-cost option in place of well-developed RRT services in LMICs but as a component of integrated and patient-centred care for those with the disease [10, 51]. This study noted that participants had not been extensively engaged in discussions regarding end-of-life care. An improvement in the provision of information on prognosis and available support is a key strategy in enhancing the utilisation of comprehensive conservative care, including palliative care [10]. Indeed, the limitations around efficient renal services in LMICs cannot be understated, including unavailability of appropriately-trained clinicians to deliver specialist care as required. Unavailability of an established palliative care pathway for the management of ESKD means that death could not even be managed in a more ‘dignified’ way for those who could not benefit from RRT. Yet, this study brings to the fore the longing need for palliative care as a potential treatment choice that could be made by individuals with ESKD and their families in Ghana. It may well be that with improvements in access and dialysis outcomes, patients’ views might be completely different. However, palliative care services should be developed for patients with multiple comorbidities or at the end of their lives in whom dialysis poses a risk of being a futile therapy [14]. Timing for initiating a discussion on palliative care is essential as participants considered it only after being on haemodialysis for a while.

The support of informal caregivers is instrumental in decisions regarding the treatment of patients with ESKD [52, 53]. Indeed, informal caregivers of patients with ESKD have their usual routines disrupted in many unexpected ways as a result of the disease, including having to accompany them to the renal centre and dealing with untoward psychological responses of patients to the disease and its treatment such as depression [53]. Yet, informal caregivers, sometimes, pressurise patients with ESKD to initiate and sustain RRT, with the belief that patients could get better with time, especially when patients are relatively young [25, 54]. Our study found that, in some instances, informal caregivers did everything in their power to ensure that the patient is sustained on treatment for as long as possible. The realities of living with the disease, however, led to a change of perspective and a preference for palliative care services.

### Study strengths and limitation

The phenomenological approach employed has given voice to those confronting mortality daily in an LMIC setting as a result of ESKD. Although the findings cannot be generalised, like all other qualitative study approaches, the study has raised insightful issues that could be explored further in other renal centres within the country and other similar settings. It paves the way for discussions about palliative care for ESKD to begin across renal centres within Ghana and other similar settings.

## Conclusions

This study has shown that individuals with ESKD or their informal caregivers would consider palliative care services, if available. Indeed, as has always been advocated, primary prevention of ESKD, including meticulous management of ESKD precursor conditions is the way to go, especially in resource-constrained settings. Notwithstanding, a reasonable number of individuals who have already developed ESKD stand to benefit from integrated renal services, including palliative care. Seeking the opinions of clinicians in such settings would be a way to bring their perspectives about palliative care to the fore.

## Supplementary information

**Additional file 1.** Interview Guide

## Data Availability

Data can be made available upon reasonable request to the corresponding author so long as it complies with regulations of the institutional review board.

## Abbreviations

ESKD: End-stage kidney disease
LMICs: Lower- and middle-income countries
RRT: Renal replacement therapy
