# Supplementary material for: Perceptions of patients with end-stage kidney disease (ESKD) and their informal caregivers on palliative care as a treatment option: a qualitative study
Source: BMC Palliat Care. 2020 Aug 20;19:133. doi: 10.1186/s12904-020-00640-y (PMC7441556; doi:10.1186/s12904-020-00640-y)
Supplement: Supplementary file 1 — Additional file 1. Interview Guide [file 12904_2020_640_MOESM1_ESM.docx]

# **Interview Guide**

Study title: Perceptions of patients with end-stage kidney disease (ESKD) and their informal caregivers on palliative care as a treatment option: a qualitative study

## **Part A: Perceptions of individuals with ESKD (patient participants)**

1. How did you know that you have ESKD?
2. What belief do you have on this illness?
3. What were your feelings and thoughts when you were first told you have ESKD?
4. Do you have any spiritual, social and psychological values that help you to cope with this condition?
5. Do you consider your condition as a life-threatening illness? If yes, what factors make you consider it as such?
6. What treatment options do you know about your illness?
7. Will you accept palliative care as an option when you realise that you are not thriving on dialysis? What factors will inform your choice?
8. What are your perceptions of life and dying? Do you see them as normal processes?
9. How do you see your family’s reaction to your treatment?
10. Are there any support systems to help you and your family to cope with the condition?

## **Part B: Perceptions of informal caregivers**

1. How do you understand your relative’s condition?
2. How did you get to know about the diagnosis?
3. Do you experience stress or challenges in caring for your relative?
4. Do you see the condition to be life-threatening? If yes, what factors make you consider it as such?
5. What treatment options do you know about this disease?
6. What is your expectation of treatment that takes care of your relatives’ physical, psychological, spiritual and social aspect of his/her health?
7. Will you accept palliative care as an option when you realise that your relative is not thriving on dialysis? What factors will inform your choice?
